# Cardiac Filaminopathies: Illuminating the Divergent Role of Filamin C Mutations in Human Cardiomyopathy

**DOI:** 10.3390/jcm10040577

**Published:** 2021-02-04

**Authors:** Matthias Eden, Norbert Frey

**Affiliations:** 1 Department of Internal Medicine III, University of Heidelberg, 69120 Heidelberg, Germany; matthiaseden@web.de; 2 German Centre for Cardiovascular Research, Partner Site Heidelberg, 69120 Heidelberg, Germany

**Keywords:** filamin C, cardiomyopathy, gene mutations

## Abstract

Over the past decades, there has been tremendous progress in understanding genetic alterations that can result in different phenotypes of human cardiomyopathies. More than a thousand mutations in various genes have been identified, indicating that distinct genetic alterations, or combinations of genetic alterations, can cause either hypertrophic (HCM), dilated (DCM), restrictive (RCM), or arrhythmogenic cardiomyopathies (ARVC). Translation of these results from “bench to bedside” can potentially group affected patients according to their molecular etiology and identify subclinical individuals at high risk for developing cardiomyopathy or patients with overt phenotypes at high risk for cardiac deterioration or sudden cardiac death. These advances provide not only mechanistic insights into the earliest manifestations of cardiomyopathy, but such efforts also hold the promise that mutation-specific pathophysiology might result in novel “personalized” therapeutic possibilities. Recently, the FLNC gene encoding the sarcomeric protein filamin C has gained special interest since FLNC mutations were found in several distinct and possibly overlapping cardiomyopathy phenotypes. Specifically, mutations in FLNC were initially only linked to myofibrillar myopathy (MFM), but are now increasingly found in various forms of human cardiomyopathy. FLNC thereby represents another example for the complex genetic and phenotypic continuum of these diseases.

## 1. Introduction

Human cardiomyopathies in general can be classified into primary and secondary cardiomyopathies. Within this classification, primary cardiomyopathies can be subdivided into pure genetic forms like hypertrophic cardiomyopathy (HCM), arrhythmogenic right ventricular cardiomyopathy (ARVC), and left ventricular non-compaction cardiomyopathy (LVNCM) as well as Ion channel, conduction, and storage disorders. Dilated cardiomyopathies (DCM) as well as restrictive cardiomyopathy (RCM) are categorized in to mixed primary cardiomyopathies, since a potential genetic etiology explains only a part of these clinical entities [1,2,3,4,5].

**Figure 1:**
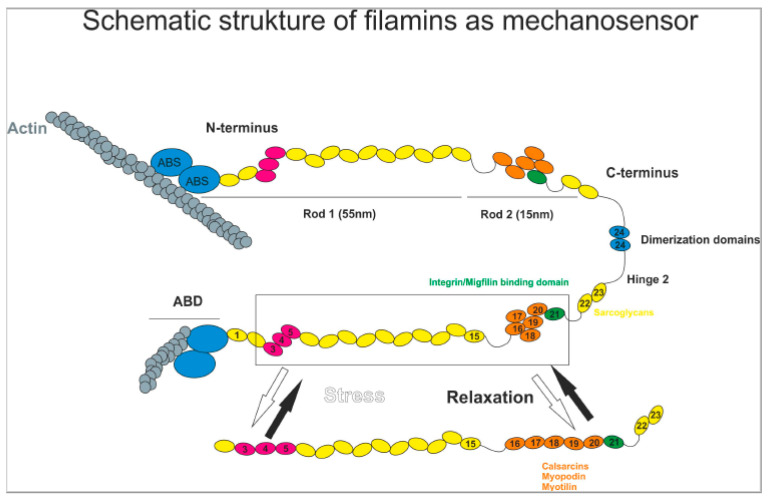
Schematic structure of filamins as mechanosensor.

As it has been previously described for filamin A (FLNA) and B (FLNB), filamin C (FLNC) is also recognized as an important structural crosslinker of actin rods at the sarcomeric z-disc of both cardiac and skeletal muscle [6]. Moreover, all three filamin variants reveal high sequence similarities indicating similar cellular functions. While FLNA and FLNB are ubiquitously expressed, FLNC is predominantly enriched in cardiac and skeletal muscle. Of note, dimerization of two identical filamins through their Ig-like domains 24 is crucial for correct filamin function (Figure 1) [4,7]. For all three filamins, a subcellular localization at the sarcomeric z-disc, intercalated discs, cell-membranes, and myotendinous junctions has been described. It is speculated that, due to their structural characteristics, in particular filamin A and filamin C also can serve as a nodal point for sarcomeric mechanotransduction in different muscle cells [7,8].

Schematic structure of Filamins binding and cross-linking F-actin via N-terminal Actin Binding Domains (ABD; blue) containing Actin Binding Sites (ABS). Distinct regions within rod 1 (R3–5; violet) and rod 2 (R16–21) are prone to spring-like conformational changes. Domains highlighted in orange are possible interaction sites for z-disc proteins, domain 21 (green) represents the possible interaction site with integrins. Domains R22–23 interact with sarcoglycans (yellow). The proposed model shows that contractile force and deformation of actin networks induce conformational changes of both filamin dimers. Subsequently, some binding partners are able to interact with exposed binding sites under mechanical stress, whereas some will rather dissociate under conformational change [7,8].

Filamin C was first reported to be associated with various forms of skeletal myopathy (i.e., MFM) [7]. The encoding *FLNC* gene consists of 48 coding exons and is located on chromosome 7q32–35. Two isoforms (one shorter isoform, lacking exon 31 and predicted to be less flexible) have been partially characterized so far. The shorter Isoform is thought to be expressed 3.5 times higher in skeletal than cardiac muscle, whereas the longer filamin C isoform seems less abundant in cardiomyocytes under basal conditions but is rapidly induced upon cardiac stress. More than 90 potential binding partners for filamin C have been denoted in the current literature [6,7]. At sarcomeric z-discs, filamin C interacts with various proteins partially linked to inherited cardiomyopathies like calsarcins (Involved in HCM [9,10,11]), myopalladin (linked to RCM [12]), cypher (linked to ARVC and DCM [13]), actin (linked to DCM [14]), myotilin, myopodin, and others. Moreover, filamin C binds to the sarcolemma via integrin-1β and sarcoglycan-delta (known as part of the muscular dystrophin complex) [15,16]. Filamin C can be cleaved by the protease calpain in order to differentially regulate the sarcoglycan-filamin interaction.

In mice, loss of *FLNC* function leads to diverse results. Whereas partial FLNC −/− mice, expressing a truncated filamin C by deletion of exons 41–48, show a severe muscular phenotype, leading to lethality due to respiratory failure, before birth, they displayed no obvious cardiac defects [17]. In contrast, a recent publication stressed the crucial role for cardiac filamin C in mice, analyzing multiple, complete FLNC knockout mouse models [18]. In contrast to the partial *FLNC* knockout, they generated conventional and heart restricted knockouts in which Cre-mediated deletion of the *FLNC* region between exons 9 and 13 resulted in subsequent frameshift of *FLNC* and, thereby, complete loss of the protein. Since these global and heart restricted FLNC −/− mice were embryonic lethal, additional inducible heart restricted FLNC knockout mice were generated by crossing *FLNC*-floxed mice with αMHC-MerCreMer mice [18]. Strikingly, these mice developed rapid progressive dilated cardiomyopathy that already occurred after 1 week knockout induced by tamoxifen treatment [18]. In humans, more than 325 unique sequence variants in *FLNC* are known (mainly affecting the longer isoform NM_001458.5), and not always resulting in distinct cardiac phenotypes in human cardiomyopathy [4]. It remains obscure why, in mutant carriers, cardiomyopathies are not accompanied by clinically overt skeletal muscle myopathies.

## 2. Filamin C Mutations Reveal a Distinct Phenotype of Human Dilated Cardiomyopathy (Dcm) with Increased Risk of Sudden Cardiac Death

DCM is one major cause for terminal heart failure ultimately leading to requirement of cardiac transplantation, left ventricular assist devices, and/or sudden cardiac death. Genetic variants cover more than 40 known genes with encoded proteins spanning a large variety of different cellular compartments [3]. Truncating *FLNC* mutations (stop or frameshift etc.) seem to be enriched in DCM patient cohorts compared to healthy individuals, while the overall prevalence of *FLNC* variants only ranges from 1% to 4.5% in different publications [4]. Interestingly, and consistent with other genetic variants found in cardiomyopathy, *FLNC* variants found in human DCM do not come along with concomitant myofibrillar myopathy. The group of Ortiz-Genga published an analysis in 2016, where 23 new truncating variants of *FLNC* were found in a DCM cohort and they reported that these gene variants were all absent in more than 1000 individuals with HCM, indicating a unique genotype phenotype correlation [19]. Surprisingly, all these patients showed no filamin aggregates in cardiac immunohistological stainings, which normally denotes a typical phenotypic feature of filamin associated MFM.

In their data set, FLNC-DCM phenotypes show marked LV-dilation and systolic dysfunction, a high degree of myocardial fibrosis (assessed by CMR and biopsies) and associated conduction abnormalities (i.e., T-Wave changes and low voltage QRS criteria in ECG recordings). One might speculate that a high degree of myocardial fibrosis and the observed conduction abnormalities in surface ECG could explain a significantly higher risk for ventricular arrhythmias (>80%) and sudden cardiac death in *FLNC* mutation carriers. Judging these typical findings, filaminopathies share some analogy to cardiac laminopathies [20,21] and in particular to desmin-related cardiomyopathies [22]. Mutations in the intermediate filament protein desmin can typically result in formation of large protein aggregates and thereby are linked to dilated cardiomyopathy [23], restrictive cardiomyopathy [24], arrhythmogenic right ventricular cardiomyopathy [25], and rarely HCM [26].

In support of these concepts, Begay et al. reported similar findings in *FLNC* truncated mutation carriers and their DCM cohort. They also speculated about a phenotypic RV involvement (seen in around 38%) and excessive fibrosis deposition assessed by electron microscopy pictures of RV-tissue. Moreover, they saw a biventricular myocardial fibro-fatty infiltration and redistribution of cell-cell junction proteins, a feature that is also typically seen in arrhythmogenic right ventricular cardiomyopathy (ARVC). These results also indicate a potential phenotypic “overlap” of DCM and ARVC in some *FLNC* mutation carriers. Since, unlike desminopathies, no protein aggregates were found in several studies, one potential mechanism of truncated *FLNC* variants affecting cardiac phenotypes is believed to be haploinsufficiency rather than storage myopathy, with reduced protein contents seen in Western blot analysis of affected individuals [27]. Whereas this proposed haploinsufficiency seems to result in late onset DCM beyond the age of 40, biallelic *FLNC* mutations (one missense (318 C > G), one stop gaining (2971 C > T)) were reported to potentially cause severe congenital dilated cardiomyopathy requiring early heart transplantation [28].

## 3. Filamin C Mutations in Arrhythmogenic Cardiomyopathy

Arrhythmogenic right ventricular cardiomyopathy (ARVC) is a genetic disorder that is diagnosed by clinical criteria affecting mainly the right ventricle and the conduction system [29,30]. In 50% of cases, the underlying genetic variant is known and mainly affecting desmosomal genes (*PKP2, DSP, JUP, DSG2 and DSC2*), genes at the area composite (*CTNNA3, CDH2*) and rarely non-desmosomal genes (*DES, LMNA, PLN, RYR, TGFB3, TTN, SCN5A, TMEM43*) [31,32]. Mutation carriers show a fibro-fatty infiltration of right ventricular myocardium and are affected by a high incidence of life-threatening arrhythmias and sudden cardiac death as well as by progressive dilation and dysfunction of the right ventricle itself [19,33,34]. Very recently, truncating *FLNC* variants were also linked to patients fulfilling ARVC criteria and with excluded genetic variants in all common ARVC genes [34]. Of note, truncating *FLNC* mutations seem to be a rather rare observation in ARVC cohorts (1%). One described genetic variant was a loss of function mutation in exon 40, the other resulted in a *FLNC* frameshift in exon 48. Interestingly, immunohistological analysis revealed altered desmosomal protein localizations but no protein aggregate accumulation in mutation carriers. Although index patients showed no signs of left-ventricular involvements, it remains unclear if these *FLNC* variants clearly are linked to an isolated ARVC phenotype.

## 4. Missense Filamin C Mutations Can Result in Human Hypertrophic Cardiomyopathy

Hypertrophic cardiomyopathy (HCM) is also a genetic disorder mainly affecting genes encoding for of sarcomeric proteins [3]. The disease is characterized by excessive and sometimes asymmetric thickening of the myocardium in the absence of afterload increasing conditions like arterial hypertension or valvular heart disease (i.e., aortic stenosis). Hypertrophic cardiomyopathy has an autosomal dominant inheritance with several hundred mutations in more than 30 genes reported so far. *MYH7, MYBPC, MYL3, TPM1,* and *TNNT2* are the most frequently mutated genes, accounting for more than 70% of all cases. *FLNC* missense mutations (mainly localized in the ROD2 domain important for cell signaling and interaction to calsarcin, synaptopodin, and nexilin at the sarcomeric z-disc; [4,35]) are believed to explain up to 10% of HCM phenotypes from patients in which common mutations in main sarcomeric genes were excluded [36]. Unlike histological findings in other cardiomyopathies, Valdes-Mas et al. also reported the formation of large mutated filamin C protein aggregates (in patients in vivo and in cell culture expressing mutated *FLNC* variants in vitro) as well as myofibril disarray and fibrosis, but again in the absence of overt skeletal myopathy [36]. Comparable to clinical courses observed in *FLNC* associated DCM and ARVC patients, HCM individuals and families expressing *FLNC* missense mutations seemed to be more prone to ventricular arrhythmias and sudden cardiac death. Mechanistically, it is speculated that, unlike truncating mutations in DCM and ARVC, missense mutations lead to loss of function phenotypes in HCM, although the precise consequences of *FLNC* missense mutations remain unexplained [36]. In a recent screen in HCM cohorts, Gomez et al. revealed that most of the found *FLNC* variants were associated with mild forms of HCM and showed reduced penetrance [37]. Beyond in contrast, one has to take into account that various other publications did not observe an excess of missense variants in HCM cohorts compared to controls, questioning the real relevance of *FLNC* sequence variants in this particular cardiac disease [38,39].

## 5. Filamin C Mutations in Restrictive Cardiomyopathy (RCM)

Restrictive cardiomyopathy is a very rare primary cardiomyopathy, according to current American Heart Association (AHA) classification, with a rather poor clinical prognosis [40]. RCM is mainly characterized by impaired diastolic function and enlarged cardiac atria, leading to diastolic heart failure, atrial fibrillation (AF), and valvular regurgitation due to severe anular dilation. Few clearly inherited RCM forms are published, and the underlying genetic mutations have only been rudimentarily characterized. Affected genes include MYH7, alpha-actin, as well as troponin T (TNT) and troponin I (TNI) subunits [41]. In 2017, Tucker et al. found a novel *FLNC* mutation in Exon 5 in a family of RCM (pV2297M) resulting in diminished sarcomeric localization, but again without protein aggregate formation [41]. In this publication, the authors speculate that like in HCM, rather “loss of function” and not haploinsufficiency explains the phenotype of the assessed *FLNC* genotype variants. 

**Figure 2:**
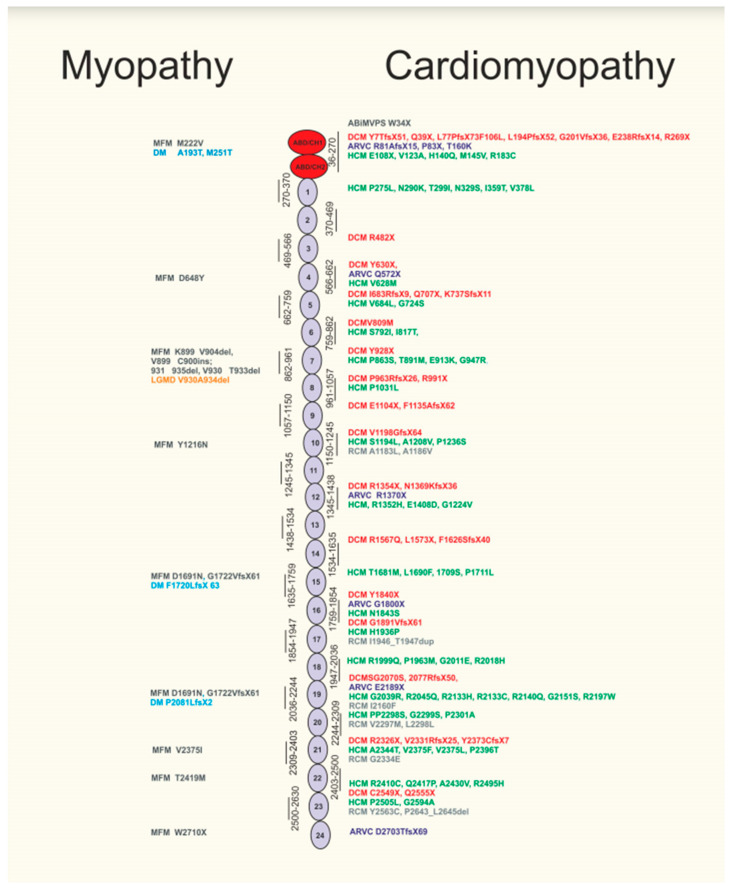
Summary of FLNC sequence variants in relation to disease phenotypes.

Overall, *FLNC* mutations can be also regarded as a potential target for newborn genetic testing for myopathy and cardiomyopathy (known sequence variants related to their phenotypes summarized in Figure 2) [42].

## 6. Filamin C Mutations in Mitral Valve Prolapse Syndrome

Very recently, a novel truncating mutation of FLNC (c201G > A; pTrp34) has also been linked to a special familiar form of arrhythmogenic bileaflet mitral valve prolapse syndrome (ABiMVPS) presenting with a combination of mitral-valve prolapse and associated electrophysiological alterations [43]. This finding, although it describes only one family pedigree, seems rather plausible since filamin A mutations have already been reported to cause similar mitral valve pathologies [7].

This illustration summarizes the structure of FLNC, showing its two calponin homology and actin binding domains (ABD/CH1 and ABD/CH2), Ig-like domains 1–24. Currently known mutations in FLNC gene are mapped to the protein structure and correlated to the phenotype of various skeletal muscle myopathies (MFM, distal myopathy (DM) and limb-girdle muscular dystrophy (LGMD)) on the left, whereas correlation with various forms of human cardiomyopathy (ABiMVPS, DCM, HCM, ARVC, RCM) phenotypes are displayed on the right (adapted from Mao et al. [7]).

## 7. Conclusions

Although it remains vague the precise mechanisms of how FLNC mutations and subsequent protein alterations affect different and partially overlapping cardiac phenotypes, it becomes increasingly clear that *FLNC* variants are found in and are associated with various forms of human cardiomyopathies. In particular in DCM, RCM, and ARVC cohorts, existing data suggests that *FLNC* mutations can affect cardiac phenotypes and even indicate patients at increased risk. Comparable to human laminopathies or desminopathies, filaminopathies seem to characterize a distinct group of electrically less stable cardiomyopathy patients. As *FLNC* mutations appear to predispose for arrhythmogenic events and sudden cardiac death in several cardiomyopathy entities, *FLNC* mutation might be an additional criteria for clinical decision making that favors early ICD implantation in cardiomyopathy. In particular, since truncating variants of *FLNC* seem to be more frequently found in patients with sudden cardiac death that *FLNC* variant carriers with missense mutations, this emphasizes a potential need for genetic testing of individuals and families [35]. This is further supported by the notion that the complex genetic heterogeneity, including resulting haploinsufficiency or loss of function variants, affects other phenotypic attributes like chamber hypertrophy and dilation. 

For a final judgement if FLNC sequence variants play a clear role in hypertrophic cardiomyopathy, bigger cohorts have to be analyzed in detail and filaminopathies have to be further characterized mechanistically.

## Data Availability

Not applicable.
